# Aseptic revisions and pulmonary embolism after surgical treatment of femoral neck fractures with cemented and cementless hemiarthroplasty in Germany: an analysis from the German Arthroplasty Registry (EPRD)

**DOI:** 10.1186/s10195-023-00689-4

**Published:** 2023-02-22

**Authors:** Dominik Szymski, Nike Walter, Paula Krull, Oliver Melsheimer, Alexander Grimberg, Volker Alt, Arnd Steinbrück, Markus Rupp

**Affiliations:** 1 Department of Trauma Surgery, University Medical Centre Regensburg, Franz-Josef-Strauss-Allee 11, 93053 Regensburg, Germany; 2 Deutsches Endoprothesenregister gGmbH (EPRD), Berlin, Germany; 3 Orthopädisch Chirurgisches Kompetenzzentrum Augsburg (OCKA), Augsburg, Germany

**Keywords:** Femoral neck fracture, Hemiarthroplasty, Embolism, Revision, Arthroplasty registry, Cementing

## Abstract

**Background:**

Femoral neck fractures (FNF) are among the most common fractures in Germany and are often treated by hemiarthroplasty (HA). The aim of this study was to compare the occurrence of aseptic revisions after cemented and uncemented HA for the treatment of FNF. Secondly, the rate of pulmonary embolism was investigated.

**Methods:**

Data collection for this study was performed using the German Arthroplasty Registry (EPRD). HAs after FNF were divided into subgroups stratified by stem fixation (cemented vs uncemented) and paired according to age, sex, BMI, and the Elixhauser score using Mahalanobis distance matching.

**Results:**

Examination of 18,180 matched cases showed a significantly increased rate of aseptic revisions in uncemented HA (*p* < 0.0001). After 1 month 2.5% of HAs with uncemented stems required an aseptic revision, while 1.5% were reported in cemented HA. After 1 and 3 years’ follow-up 3.9% and 4.5% of uncemented HA and 2.2% and 2.5% of cemented HA needed aseptic revision surgery. In particular, the proportion of periprosthetic fractures was increased in cementless implanted HA (*p* < 0.0001). During in-patient stays, pulmonary emboli occurred more frequently after cemented HA [0.81% vs 0.53% in cementless HA (OR: 1.53; *p* = 0.057)].

**Conclusion:**

For uncemented hemiarthroplasties a statistically significantly increased rate of aseptic revisions and periprosthetic fractures was evident within a time period of 5 years after implantation. During the in-hospital stay, patients with cemented HA experienced an increased rate of pulmonary embolism, but without statistically significant results. Based on the present results, with knowledge of prevention measurements and correct cementation technique, cemented HA should be preferred when using HA in the treatment of femoral neck fractures.

*Trail registration*: The study design of the German Arthroplasty Registry was approved by the University of Kiel (ID: D 473/11).

*Level of Evidence*: Level III, Prognostic.

## Introduction

Fractures of the femoral neck (FNF) are among the most common fractures in the German population, with an annual number of over 81,000 fractures and an incidence of 120.2 per 100,000 inhabitants. Both an increase of 23% between 2009 and 2019 and a high incidence in the population over 70 years of age (508.2 fractures per 100,000 population/year) underline the relevance of these fractures for the health care system [1–3]. The increasingly aging society in Germany and the higher prevalence of osteoporosis in old age depict two driving factors of the increasing prevalence of this fracture type [1, 4]. In 1997, Gullberg et al. identified a prognostic approach for estimating the worldwide prevalence of FNF. A doubling from 1990 to 2025, and a further doubling to 2050 was predicted [5]. Based on the epidemiological data in Germany, a significant increase in FNF rate between 1.0 and 2.3% per year can be expected [1, 3, 6].

In FNF, surgical treatment is the treatment of choice in almost all cases. This ensures rapid restoration of function and mobility [7]. In older patients with evident advanced joint degeneration, treatment by (partial) joint replacement is the gold standard. The advantage of a partial joint replacement [hemiarthroplasty (HA)] is the lower invasiveness and consequently shorter operation time combined with less blood loss and a lower complication rate. Total hip arthroplasty, on the other hand, is described as resulting in better hip joint function and better quality of life [7–9]. However, patients demonstrate a significantly increased mortality rate within a minimum of 8 years follow-up. Contrariwise, a higher implant survival rate was reported [10, 11].

Current data from Germany show that the vast majority of FNF are treated by HA. In most cases, the femoral fixation option using cementation is used [3]. Okike et al. already showed that fewer aseptic revision procedures need to be performed after cemented anchorage of partial arthroplasties, while no significant differences between the two fixation techniques were found for both in-hospital mortality and 1-year mortality in the USA [12]. Initial signs of increased in-hospital mortality in cemented HA are apparent in analyses of large registry data. Thereby, in recent literature the overall mortality rate was reported to range from 16% to 33% within the first postoperative year [13, 14]. However, in Germany or Europe no sufficient and reliable data on aseptic revisions after cemented and uncemented HA are available. Regarding mortality, the development of a pulmonary embolism after cemented stem implantation is a controversial issue with no available data to answer this important question.

Using the German Arthroplasty Registry (EPRD), the aim of the present study was to compare aseptic complications of cemented and uncemented hemiarthroplasties after treatment of femoral neck fractures. In addition, the occurrence and etiology of aseptic complications, and the incidence of pulmonary embolism during the primary in-patient stay were analyzed.

## Methods

### Data collection

Since 2012, implantations of arthroplasties have been documented in the German Arthroplasty Registry [“Endoprothesenregister Deutschland” (EPRD)] in collaboration with the statutory health insurance funds in Germany (AOK Bundesverband GbR, Verband der Ersatzkassen e.V vdek), the German Medical Technology Association (BVMed), and several participating hospitals. By 2020, more than 1.6 million procedures had been reported to the registry, covering approximately 70% of all hip and knee arthroplasties [15]. By including the two participating health insurance associations (AOK-B, vdek), approximately 65% of the German population was included in the data collection, and the information provided by hospitals and surgeons could be cross-validated. Surgical revisions registered in the EPRD are followed up based on insurance billing data, even if performed in a hospital not participating in the arthroplasty registry. With the exception of procedures performed outside Germany, this algorithm ensures tracking of patients insured by these companies [16].

For the classification and identification of diagnoses and procedures, the German versions of the International Classification of Procedures in Medicine (ICPM), the “Operation and Procedure Code” (OPS) 301 system, and the 10th International Classification of Diseases (ICD-10) were used.

### Patients

**Fig. 1:**
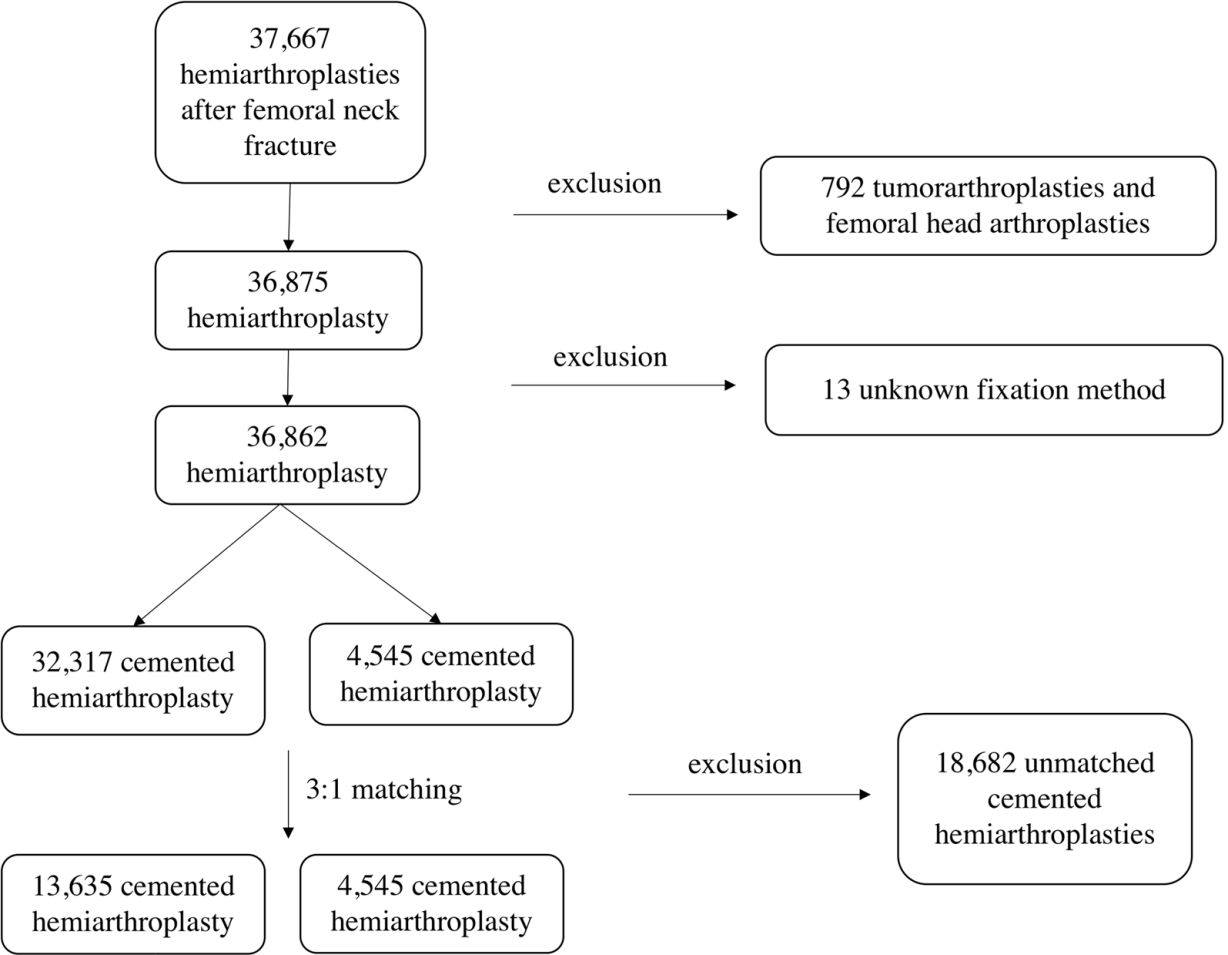
Flow-chart of the study population with patient inclusion and exclusion criteria

All patients with HA after FNF were included in the present analysis of the German Arthroplasty Registry (EPRD). Patients were divided into cases with cemented and uncemented femoral fixation of partial hip joint replacements and paired according to age, sex, body mass index (BMI), and Elixhauser comorbidity score (the van Walraven variant) using Mahalanobis distance matching in a 3:1 ratio. The Elixhauser comorbidity score is an index that pools a variety of comorbidities of different organ systems and entities [17]. In addition to comorbidities, all other billing diagnoses are recorded in the arthroplasty registry, which were used to determine influencing factors. Patients who were not treated with HA, and for whom no FNF was coded as the main diagnosis were excluded. Patients in whom no statement could be made regarding stem anchorage were also excluded from the data collection. Likewise, the use of special implants such as tumor prostheses and femoral head-only prostheses were excluded (Fig. 1).

### Statistical analysis

The data were analyzed to determine aseptic revision and pulmonary embolism rates for cemented and uncemented hemiarthroplasties after femoral neck fractures in Germany. To account for bias in the selection of patients for a particular treatment (cemented or uncemented hemiarthroplasty), matching of patients was performed using the variables of sex, age at the time of surgery, and the van-Walraven weighted version of the Elixhauser comorbidity score [17] and, if the information was available, the patient’s BMI.

The statistical program R (R Foundation for Statistical Computing, Vienna, Austria) was used to perform the statistical analysis. For postmatching statistical analysis, Kaplan–Meier estimates were calculated, log-rank tests were performed, and hazard ratios were calculated for the matched data. Because not every revision procedure was documented directly in the EPRD (for example, if that revision occurred in a hospital not participating in the EPRD), a weighted Kaplan–Meier estimator was used to analyze the principle-specific revision probabilities [18, 19]. Here, all HA that were changed during the follow-up period but for which the reason for change was unknown were excluded from the analysis. Arthroplasties that were changed during the follow-up period with a known revision reason were weighted by 1. In order to maintain the relations within the data set, all arthroplasties with no revision surgery during the follow-up period were weighted by the respective percentage of revisions without known reason. The significance level was set at α = 0.05.

## Results

A total of 36,862 patients with FNF and treatment by HA were identified in the German Arthroplasty Registry (EPRD) in this study and used for matching. After matching using age, sex, Elixhauser comorbidity score, and BMI, 18,180 patients were included in further data analysis (Table 1).

**Table 1:** Anthropometry and risk factors before and after matching of the patient cohort with cemented and uncemented hemiarthroplasty after femoral neck fracture

	Uncemented HA	Cemented HA	*p*-value
Number (*n*)	4545	13,635	
Age (years)	82.27 ± 8.42	82.44 ± 7.95	*p* = 0.206
Sex (female) *n* (%)	3062 (67.4)	9189 (67.4)	*p* = 0.993
Elixhauser comorbidity score	8.35 ± 7.86	8.38 ± 7.75	*p* = 0.838
Body mass index in kg/m^2^	25.01 ± 4.24	24.97 ± 4.18	*p* = 0.615

**Fig. 2:**
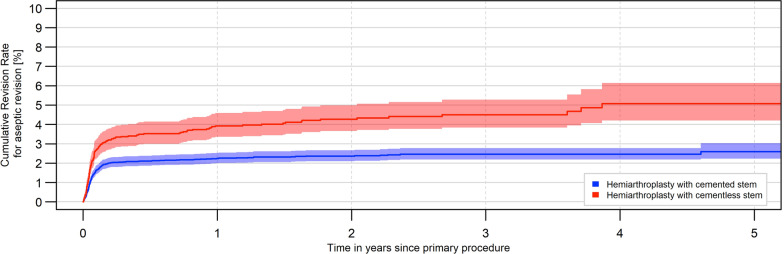
Development of aseptic revisions within the first 5 years after implantation of cemented or uncemented hemiarthroplasty for the treatment of a femoral neck fracture (log-rank test: *p* < 0.0001)

**Fig. 3:**
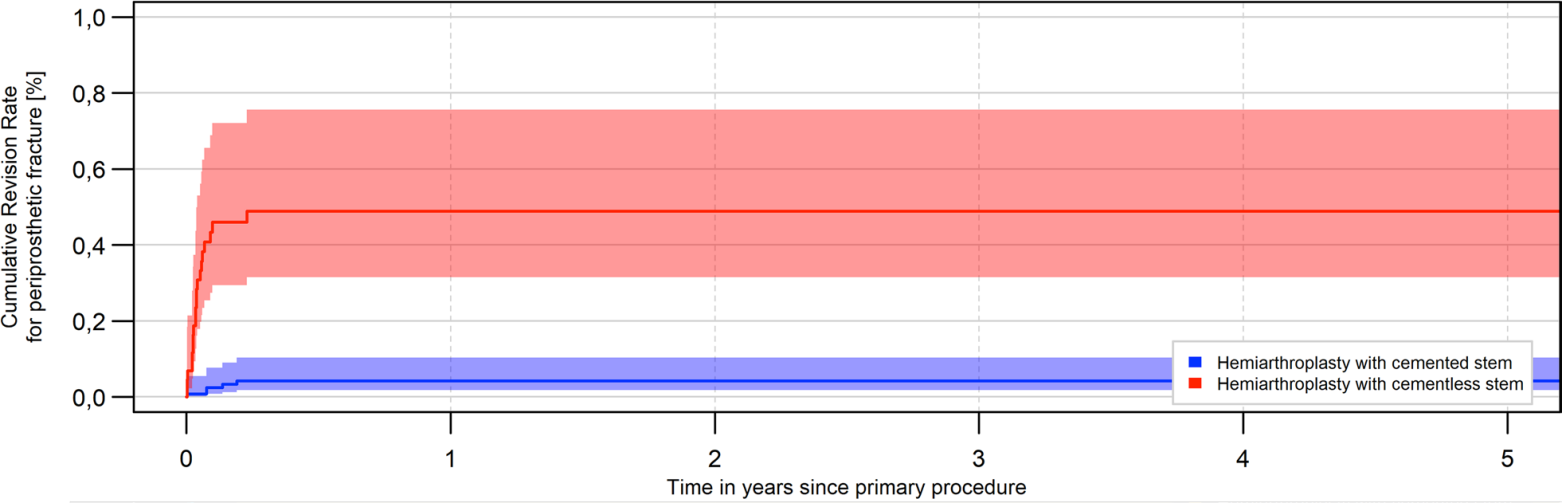
Development of periprosthetic fractures within the first 5 years after implantation of a cemented or uncemented hemiarthroplasty for the treatment of a femoral neck fracture (log-rank test: *p* < 0.0001)

A statistically significant increase of aseptic revisions was reported for the uncemented HA (*p* < 0.0001). The proportion increased from 2.5% after 1 month to 3.9% after 1 year and 4.5% after 3 years, while 1.5% of cemented HA had to be replaced after 1 month, 2.2% after 1 year and 2.5% after 3 years due to aseptic events. The hazard ratio was 0.56 for stems fixated with bone cement in terms of a reduced risk of aseptic revision (95% CI 0.46–0.68) (Table 2; Fig. 2). The most frequent reason for an aseptic revision of cementless stem was periprosthetic fracture (52.2%) and dislocation (20.1%). For cemented stem fixation of HA, the most common reported reason for revision was dislocation (44.8%). Periprosthetic fractures were only registered in 5.3% of cases after cemented HA. Between uncemented and cemented HA a statistically significant difference was found with regard to periprosthetic fractures (*p* < 0.0001) (Fig. 3).

**Table 2:** Percentage of aseptic revisions after femoral neck fracture treated with cemented and uncemented hemiarthroplasty

	Aseptic change in % [95% confidence interval]					
	1 month	3 months	6 months	1 year	2 years	3 years
Uncemented HA	2.5 [2.1; 3.0]	3.3 [2.8; 3.9]	3.5 [3.0; 4.1]	3.9 [3.4; 4.6]	4.3 [3.7; 5.0]	4.5 [3.8; 5.3]
Cemented HA	1.5 [1.3; 1.7]	2.0 [1.8; 2.3]	2.1 [1.9; 2.4]	2.2 [2.0; 2.5]	2.4 [2.1; 2.7]	2.5 [2.2; 2.8]

With cemented HA, pulmonary embolism occurred in 0.81%, while 0.53% of patients receiving uncemented prostheses experienced embolism during the in-patient stay. There was an increased risk of pulmonary embolism after cemented stem fixation with an odds ratio of 1.53 (95% CI 0.98–2.50). However, this difference was not statistically significant (*p* = 0.057).

## Discussion

This registry study investigated the occurrence of aseptic complications, which led to revision surgery, and the prevalence of postoperative pulmonary embolisms of cemented and uncemented HAs after FNF with a follow-up of up to 5 years by analyzing the German Arthroplasty Register (EPRD). A prospective investigation of a 3:1 matched population of patients with cemented and uncemented HA was obtained for the treatment of FNF. In addition, cross-validation and precise follow-up data acquisition was possible through data input by EPRD registered hospitals and data provided by health insurance companies.

Femoral stem fixation is controversial in the surgical management of FNF. In 2010, the working group around Parker et al. was able to clearly demonstrate the advantages of cemented treatment with faster mobilization and reduction of postoperative pain by means of a systematic review [20]. In addition to a reduction in postoperative pain and faster mobilization of patients after use of a partial arthroplasty with a cemented stem, a lower proportion of stem sintering was also described in the literature [20, 21]. Similarly, the proportion of aseptic revisions was significantly lower with HAs with cemented femoral stem fixation compared with the cementless alternative [12, 22–24]. Okike et al. described a significantly increased risk of aseptic revision for uncemented HA, with a hazards ratio of 1.77 (95% CI 1.43–2.19) [12]. Furthermore, a 2.1 times higher rate of revision surgeries for aseptic failure of HA were reported in an analysis of the Norwegian Hip Fracture Register [25]. The evaluation of the EPRD also demonstrated a significant difference for aseptic revisions with a hazard ratio of 0.56 for cemented partial prostheses. The replacement rate for cemented stems was 2.5% after 3 years, the same level as for uncemented HAs after 1 month. The literature cites a lower number of periprosthetic fractures with cemented stems as the main reason for the lower revision rate [22]. In particular, patients with risk factors for periprosthetic fractures, such as increased patient age, osteoporosis, and a recurrent tendency to fall benefit from cemented fixation [26]. This hypothesis could also be supported by the significant difference in periprosthetic fractures as the reason for replacement surgery in our study. While the proportion of periprosthetic fractures as a reason for revision in cemented HA was 5.2%, the proportion in the cementless variant was about tenfold higher, at 52.2%. In addition to the stabilizing effect of bone cement, the possibility of insufficient osseous integration of the cementless stem is discussed as a reason for this issue [26, 27]. Besides periprosthetic fractures, which were also reported as the main cause of aseptic revision surgery in the literature, dislocation of the prosthesis and acetabular wear were reported as important reasons for reoperations [28].

With regard to mortality, no clear advantage of a particular stem anchorage technique could be shown in previous studies over the long term [22, 24, 25, 29, 30]. However, there are some problems concerning a cemented fixation of a HA caused by side-effects of cemented fixation. Geographical differences are also noticeable in this context, with mainly cemented HA being used in Europe, whereas mainly uncemented HA are used in the USA. [3, 31]. Several studies reported an increased in-patient mortality for cemented joint replacement surgeries and demonstrated an increased mortality for cemented HA with an odds ratio of 1.64 (95% CI 1.35–2.00) within the first 48 h after surgery [30, 32]. Also, first signs of increased in-hospital mortality are apparent for cemented HA in the analysis of large register data. The theory for the increased mortality immediately after implantation is the bone cement implantation syndrome (BCIS). This occurs in up to 28% of cemented partial arthroplasties and is manifested by hypoxia, sudden loss of arterial pressure, pulmonary hypertension, and arrhythmias, potentially leading to cardiac arrest [33, 34]. However, the exact pathomechanism has not been fully elucidated, and a multifactorial cause seems most likely [34]. The frequently occurring intraoperative pulmonary emboli, that sometimes present only subclinically, seem to play a major role [35]. In 0.81% of the investigated cemented HAs in our study, a documented and clinically relevant pulmonary embolism occurred, which resulted in further diagnosis and therapy. There was no statistically significant difference (*p* = 0.057) compared with the uncemented stem anchorage variant; however, an odds ratio of 1.53 in terms of an increased risk for cemented HA was calculated. Li et al. showed a significantly increased rate of pulmonary embolism after cemented fixation in a meta-analysis [36]. Patients with pulmonary embolism subsequently show significantly increased mortality compared with patients without pulmonary embolism, both immediately postoperatively and after 1, 3 and even 6 months [37]. Preventive measures and the implementation of safety guidelines in the use of bone cement, especially in HA after femoral neck fracture, are therefore strongly recommended [38].

Despite many advantages of this study, some limitations must be mentioned. The quality of the registry depends on the quality of the information provided by the surgeons and the coding of the hospitals. Validation of the arthroplasty registry protocol with insurance and billing procedures can minimize, but not completely remove this effect.

In the analysis of pulmonary emboli, the registry is similarly dependent on correct coding and can only represent events during the first in-patient stay. However, the aim of the study was to investigate embolisms directly related to prosthesis implantation. Therefore, the period of acute in-patient treatment seems to be suitable. Another limitation is the patient follow-up period of 5 years for aseptic revisions. Due to the age of the register the investigation time is limited here.

## Conclusion

For uncemented hemiarthroplasties a statistically significantly increased rate of aseptic revisions and periprosthetic fractures was evident within a time period of 5 years after implantation. During the in-hospital stay, patients with cemented HA experienced an increased rate of pulmonary embolism, but without statistically significant results. Based on the present results, with knowledge of prevention measurements and correct cementation technique, cemented HA should be preferred when using HA in the treatment of femoral neck fractures.

## Data Availability

Data available on request.
